# Synthesis and In Vitro Evaluation of a Scandium-44 Radiolabeled Nanobody as a PD-L1 PET Imaging Probe

**DOI:** 10.3390/pharmaceutics17060796

**Published:** 2025-06-19

**Authors:** Viktoria E. Krol, Aditya Bansal, Manasa Kethamreddy, Jason R. Ellinghuysen, Daniel J. Vail, Fabrice Lucien-Matteoni, Haidong Dong, Sean S. Park, Mukesh K. Pandey

**Affiliations:** 1 Department of Radiology, Mayo Clinic, Rochester, MN 55905, USA; krol.wiktoria@mayo.edu (V.E.K.); bansal.aditya@mayo.edu (A.B.); kethamreddy.manasa@mayo.edu (M.K.); ellinghuysen.jason@mayo.edu (J.R.E.); vail.daniel@mayo.edu (D.J.V.); 2 Department of Urology, Mayo Clinic, Rochester, MN 55905, USA; lucien-matteoni.fabrice@mayo.edu (F.L.-M.); dong.haidong@mayo.edu (H.D.); 3 Department of Immunology, Mayo Clinic, Rochester, MN 55905, USA; 4 Department of Radiation Oncology, Mayo Clinic, Rochester, MN 55905, USA; park.sean@mayo.edu; 5 Department of Pharmacology, Mayo Clinic, Rochester, MN 55905, USA; 6 Mayo Clinic Comprehensive Cancer Center, Rochester, MN 55905, USA

**Keywords:** Scandium-44, immunoPET, molecular imaging, PD-L1, breast cancer

## Abstract

**Background/Objective**: Noninvasive PET imaging-based assessment of PD-L1 expression is of high clinical value for better patient selection and treatment response rates to PD-L1 immunotherapies. Due to their shorter biological half-life and faster clearance from the blood pool, radiolabeled antibody fragments are an attractive alternative for imaging than their full-length IgG counterpart. This work investigated the radiosynthesis and in vitro cell uptake of anti-PD-L1-B11-nanobody radiolabeled with ^44^Sc (t_1/2_ = 4.04 h) as an alternative to anti-PD-L1-B11-IgG, better suited for longer half-life radioisotopes such as ^89^Zr (t_1/2_ = 78.41 h). **Methods**: The proteins were conjugated with p-SCN-Bn-DTPA and radiolabeled at room temperature with ^44^Sc, achieving a radiochemical yield of a RCY of 94.8 ± 3.1% (*n* = 3) for [^44^Sc]Sc-B11-IgG and 73.6 ± 12.1% (*n* = 3) for [^44^Sc]Sc-B11-nanobody, before purification. **Results**: Significantly higher uptake in the PD-L1_+_ cells than PD-L1_KO_ cells was observed for both probes. However, high non-specific uptake, particularly of the radiolabeled B11-nanobody, was also observed which may negatively impact its potential as a molecular imaging probe. **Conclusions**: Due to the high non-specific uptake in vitro, the ^44^Sc radiolabeled nanobody was not progressed to further in vivo evaluation. These results should, however, not discourage future evaluations of other nanobody based probes radiolabeled with ^44^Sc, due to their well-matched biological and physical half-life.

## 1. Introduction

Programmed death ligand-1 (PD-L1) is an immune inhibitory molecule, working as an antigen to programed death-1 (PD-1) receptors found on T-cells. The binding of this receptor has the function of deactivating the T-cell and preventing activation of new naïve T-cells [1,2,3]. On healthy cells, this expression is necessary for protecting against dysregulated immune response [4]. However, expression of PD-L1 on cancer cells hijacks this deactivation pathway to in turn shield them from T-cell induced cell death. One therapeutic approach against this is immune checkpoint inhibition therapy which uses anti-PD-L1 antibodies to inhibit the receptor-ligand interaction and subsequently promote anti-cancer T-cell-mediated immune response [5,6,7,8,9]. This form of treatment has been demonstrated to be highly effective, but only for small subsets of patients. Unsurprisingly, the responsiveness of patients to anti-PD-L1 immunotherapies is directly correlated to the level of PD-L1 expression [10]. The current gold standard method for quantifying PD-L1 is immunohistochemistry, but this method suffers from limitations such as sampling error due to tumor heterogeneity [11]. To this end, noninvasive positron emission tomography (PET) probes are emerging as promising alternatives to image quantitatively the whole tumor volume and better qualify patients for immunotherapies [12,13,14]. In this approach, anti-PD-L1 antibodies are radiolabeled with a positron-emitting radionuclide allowing for their biodistribution and uptake to be tracked in vivo and thereby enabling molecular tumor profiling [15]. Due to the long biological half-life of full-length monoclonal antibodies, it takes several days for optimal uptake, and they, therefore, must be radiolabeled with an appropriately long-lived radionuclide, warranting the use of zirconium-89 (^89^Zr) with a half-life of 78.41 h [16,17,18]. For immunoPET probes, it is of significant interest to develop antibody fragments which maintain their specificity and affinity for the target receptor while their reduced size allows for faster clearance from the blood pool [19,20]. Previously, our group showed promising in vivo results of the in-house developed [^89^Zr]Zr-DFO-B11-anti-PD-L1 (herein, [^89^Zr]Zr-B11-IgG) compound featuring a full-length humanized IgG antibody which was able to distinguish between PD-L1 expressing and PD-L1 knockout tumors in breast cancer and an melanoma animal model [21]. However, the ~150 kDa molecular weight antibody did not show optimal uptake and signal to background ratio (SBR) until several days post-administration. In a clinical setting, the logistical challenge of requiring the patient to return for the PET scan several days after administration would likely significantly stifle widespread adoption [22]. In this work, we present the radiosynthesis and pre-clinical evaluation of [^44^Sc]Sc-DTPA-B11-anti-PD-L1-nanobody (herein, [^44^Sc]Sc-B11-nanobody), a ~15 kDa antibody fragment of the full-length B11-IgG radiolabeled with the PET radionuclide scandium-44 (^44^Sc). ^44^Sc is gaining momentum towards clinical applications due to its favorable decay properties including a 94% positron emission intensity and ‘goldilocks’ half-life of 4.04 h—filling a significant market gap between ^68^Ga (t_1/2_ = 67.7 min) and ^64^Cu (t_1/2_ = 12.7 h) for use with peptides and antibody fragments [23,24,25]. Moreover, ^44^Sc forms a direct theranostic pair with ^47^Sc (β^−^100%, t_1/2_ = 3.35 days) and is considered an excellent imaging surrogate for ^177^Lu targeted radionuclide therapy (TRT) [26,27]. The purpose of this work is to evaluate the radiosynthesis of [^44^Sc]Sc-B11-nanobody and its potential as a PD-L1 imaging probe. [^44^Sc]Sc-B11-nanobody and [^44^Sc]Sc-B11-IgG were investigated in parallel to compare the performance of the proteins while controlling for the potential effect of varying the metal-chelator complex on the performance of the antibody fragment.

## 2. Materials and Methods

### 2.1. Production and Purification of ^44^Sc

^44^Sc was produced via the ^44^Ca(p,n)^44^Sc reaction on a 16.5 MeV GE PETtrace cyclotron with the target at 30°, the beamline and beam energy degraded to ~11.7 MeV using a 0.3 mm Al degrader foil, calculated using SRIM-2013 software [28]. Two target materials, ^nat^CaCO_3_ and ^nat^CaO, were evaluated in pressed powder pellet form, with the ^nat^CaO showing better stability under increasing beam current (10–40 µA) and time (10 min–2 h). For subsequent radiolabeling studies, 100–150 mg ^nat^CaO target was irradiated for 30 min–1 h at 40 µA. Post-irradiation, the powder pellet was extracted and dissolved in 5.0 mL of 3 M HCl. For purification of ^44^Sc from the bulk Ca target, the dissolved target was passed through a two-column anion exchange purification system described previously by van der Meulen et al., [25]. Briefly, the first column contained 150 mg unbranched DGA pre-conditioned with 15.0 mL of 3 M HCl. The dissolved target was loaded, and bulk Ca^2+^ was washed from the column with 30.0 mL of 3 M HCl while the Sc^3+^ was retained on the resin. This was followed by 10.0 mL of 1 M HNO_3_ wash to remove trace metal impurities of Al and Fe, and finally elution of Sc^3+^ using 10.0 mL of 0.1 M HCl directly onto an SCX resin for preconcentration and fine purification. The SCX resin was used as purchased and ^44^Sc was eluted using 5 M NaCl/0.13 M HCl (1:1 *v*/*v*) in three fractions of 0.5 mL. The pH of the eluted ^44^Sc was adjusted to pH 5.5–6.0 for radiolabeling using 0.5 M NaOAc.

### 2.2. Quality Control of ^44^Sc

#### 2.2.1. HPGe Analysis of Radioimpurities

The presence of radioimpurities was identified using a high-purity germanium (HPGe) detector (Mirion Technologies, Atlanta, GA, USA) using a 10 µL spot of the dissolved target solution at 10 cm above the detector surface. Samples were analyzed 30–60 min after end of bombardment (EOB), and for up to two weeks post-production to more easily identify low levels of longer-lived impurities.

#### 2.2.2. MP-AES Analysis

The presence of Ca (317.933 nm), Al (396.152 nm) and Fe (371.993 nm) in the fractions eluted from the SCX column was analyzed using microwave plasma atomic emission spectroscopy (MP-AES) on an Agilent MP-AES-200 instrument equipped with SPS 4 autosampler. The measurement conditions are summarized in Table 1. Calibration curves from 0.1–10 ppm were generated through serial dilution of single-element standard solutions purchased from Agilent Technologies, Santa Clara, CA, USA. Final ^44^Sc elution fractions (3 × 0.5 mL per production) were diluted using 5% HNO_3_ and analyzed from three separate productions. Each measurement was taken in triplicate.

The authors note that in some publications related to MP-AES analysis of samples containing Ca, the use of an ionization suppressant (typically CsNO_3_) is recommend due to its low ionization energy [29,30]. This can have the effect of easily oversaturating the detector and suppressing the signal of atoms with higher ionization energy (resulting in an underestimation of their presence). During our method development, it was demonstrated the presence of Ca and Al from controlled samples prepared using standard solutions were accurately recovered when the Ca:Al ratio was between 1:1–1000:1 (1 ppm:1 ppm–100 ppm:0.1 ppm), with no added advantage of the ionization suppressant in this range (Supplementary Table S1). An ionization suppressant was, therefore, not used in the analysis of the final fractions, but would be recommended for analysis of trace metal impurities in, for example, fractions from the bulk Ca wash, where the presence of Ca is much higher.

### 2.3. Synthesis of (Anti-PD-L1) DTPA-B11-Nanobody and DTPA-B11-IgG

The full-length B11-IgG and B11-nanobody were conjugated to S-2-(4-Isothiocyanatobenzyl)-diethylenetriamine pentaacetic acid (p-SCN-Bn-DTPA) (Macrocylics, Plano, TX, USA), a bifunctional acyclic chelator for ^44^Sc, by adding a three-fold molar excess of chelator in 1X PBS at pH 9.0 and 37 °C for 30 and 60 min, respectively. The conjugation ratio was determined via matrix-assisted laser desorption ionization time-of-flight (MALDI-TOF) analysis, performed at the Mass Spectrometry Facility, School of Chemical Sciences, University of Illinois at Urbana Champaign. The conjugated antibodies/antibody fragments were purified and buffer exchanged in 0.1 M NaOAc pH 6.0 using Zeba spin desalting columns with a 7 kDa molecular weight cut-off (MWCO) (ThermoFisher Scientific, Waltham, MA, USA). The protein concentration of samples was analyzed via a Bradford assay [31] using a FLUOstar OPTIMA system (BMG Labtech, Ortenberg, Germany).

### 2.4. Radiosynthesis

For the radiosynthesis of [^44^Sc]Sc-B11-IgG, 100–150 µL (130–255 µg) of the DTPA-B11-IgG was added to 100 µL (8.1–9.2 MBq) of pH-adjusted ^44^Sc and topped with 100 µL 0.25 M NaOAc with a final pH of 5.7–6.0. For the radiosynthesis of [^44^Sc]Sc-B11-nanobody, 35–150 µL (17.5–30 µg) of the DTPA-B11-nanobody was added to 100 µL (8.2–9.3 MBq) of pH-adjusted ^44^Sc and topped with 100 µL 0.25 M NaOAc (pH 5.7–6.0). The radiolabeling reactions were performed at room temperature for 30 min. Where the radiolabeling yield was <99%, the compounds were purified using a G-25 Sephadex (PD-10) desalting column (Cytiva, Marlborough MA, USA), collecting the highest concentration fraction for further studies. The radiolabeling yield (RCY) and radiochemical purity (RCP) were determined via spotting 0.5 µL on iTLC-SG-strips in triplicate and assessed using a radio-TLC scanner (Eckert and Ziegler, Valencia, CA, USA) with 0.1 M sodium citrate, pH 5.0, as the mobile phase. The radiolabeled compound remained at the origin (R_f_ = 0) and free ^44^Sc traveled with the solvent front (R_f_ = 1).

### 2.5. SDS-PAGE

Sodium dodecyl sulfate–polyacrylamide gel electrophoresis (SDS-PAGE) and autoradiography were performed as per previously established protocol [32,33]. The radiolabeled and unconjugated versions of B11-IgG and B11-nanobody were diluted with 2× Laemmli sample buffer (1:1, *v*:*v*) (Bio-Rad laboratories, Hercules, CA, USA) with and without 10× NuPAGE sample reducing agent (Life Technologies Corporation, Carlsbad, CA, USA). For the reducing condition, the samples were reduced at 80 °C for 3 min. The reduced and non-reduced proteins were resolved by one-dimensional SDS-PAGE in 10.0% Mini-PROTEAN TGX Gel (Bio-Rad laboratories, Hercules, CA, USA) using 1× Tris–Glycine-SDS running buffer and stained using Coomassie G-250 stain (Bio-Rad laboratories, Hercules, CA, USA).

### 2.6. Autoradiography

To identify the radiolabeled proteins, autoradiography was performed on the SDS-PAGE gels after electrophoresis using a Cyclone Plus Storage Phosphor System (PerkinElmer Corporation, Waltham, MA, USA) and visualized using Image J 1.54g software.

### 2.7. Serum Stability Analysis

The stability of the radiolabeled compounds was assessed as formulated and in mouse and human sera in a 1:1 (*v*/*v*) ratio at 37 °C with light agitation (500 rpm) in a thermomixer and assessed using radio-iTLC at 2, 4, 6 and 8 h. The percentage of intact radiolabeled compound is calculated using the area under the curve (AUC) at the origin (R_f_ = 0) relative to the total AUC at R_f_ = 0 and R_f_ = 1 (free ^44^Sc).

(1) $$\%\;Intact=\frac{{AUC}_{Rf=0}}{{AUC}_{Rf=0}+{AUC}_{Rf=1}}\times 100$$

### 2.8. Cell Studies

The cell lines were developed as previously described [21,34]. Briefly, the E0771 mouse triple-negative breast cancer cell line was obtained from Robin L. Anderson at Olivia Newton-John Cancer Research Institute (Heidelberg, Australia). A PD-L1 knock-out version (E0771-PD-L1_KO_) was generated using CRISPR/Cas9 technology to obtain the PD-L1 negative control cell line. Human PD-L1 (hPD-L1) was then expressed in the E0771-PD-L1_KO_ cell line to generate the hPD-L1 positive E0771 (E0771 PD-L1_+_) cell line. For the in vitro cell uptake study, E0771-PD-L1_+_ and E0771-PD-L1_KO_ cells were plated in a 6-well plate at a concentration of ~1 × 10^6^ cells/well. After overnight culture, 5 and 30 pmol/well of [^44^Sc]Sc-B11-IgG and [^44^Sc]Sc-B11-nanobody were added to each well and incubated in a humidified CO_2_ incubator for 2 h at 37 °C. Post-incubation, the cells were washed three times with cold Hank’s buffered salt solution (HBSS), trypsinized, and counted using an automated 2480 Wizard2 gamma counter (PerkinElmer, Waltham, MA, USA). The uptake is reported as a % uptake per 1 × 10^6^ cells.

## 3. Results

### 3.1. Scadium-44 Production

In comparing the performance of the ^nat^CaO and ^nat^CaCO_3_ targets, the ^nat^CaO was successfully tested up to 40 µA for 2 h irradiation while the ^nat^CaCO_3_ target showed significant fragmentation even at 10 µA for 15 min (Supplementary Figure S1), consistent with previous reports by others [35]. For routine production and all radiolabeling experiments reported in this work, ^44^Sc was produced from a ^nat^CaO target. With this target, a maximum EOB activity of 943.13 ± 59.94 MBq (40 µA, 2 h, *n* = 3) was achieved (Table 2), with an average yield of 12.12 ± 1.70 MBq/µAh (*n* = 50).

The presence the of low-level radioimpurities ^44m^Sc (t_1/2_ = 58.61 h, E_y_ = 271.35 keV), ^47^Sc (t_1/2_ = 3.35 days, E_β-_142.6, 203.9 keV), ^48^Sc t_1/2_ = 43.7 h, E_y_ = 175.4, 983.5, 1037.5, 1312.1 keV was picked up at later time points (>1 day post EOB) once the ^44^Sc activity (E_γ_ = 1157 keV) had decayed significantly (Supplementary Figure S2). Quantitative analysis of the radioimpurities is, therefore, not reported here, but is expected to be below 1% based on analysis at similar beam parameters reported by others [36]. The widespread adoption of any radiometal is inevitably linked to its ease of production at clinically relevant scales. CaO/CaCO_3_ pressed powder targets were selected as opposed to solid Ca metal targets as these are common materials for receiving the enriched ^44^Ca material, anticipated in future work. Based on our experimental data, the predicted yield for identical experiments with the enriched material (>98% ^44^Ca vs. 2% ^44^Ca in ^nat^Ca) is expected to exceed 500 MBq/µAh.

Post-purification, the Ca, Al and Fe content in the eluted fractions was measured using MP-AES and is summarized in Table 3. As expected, the presence of Ca is higher than that of the other trace metals due to the remnant target material. Nonetheless, an average of ~10 ppm (0.01 mg/mL) Ca concentration is indicative of a highly effective bulk separation starting from ≥10,000 ppm (≥10 mg/mL).

### 3.2. Antibody and Antibody Fragment Conjugation

The nanobody fragment (B11-nanobody) of the humanized anti-PD-LI-B11 variant HC4LC4 was synthesized and characterized as previously reported [34]. Conjugation of the acyclic bi-functional chelator p-Bn-NCS-DTPA was performed as described in the methods section. The conjugation ratio (protein:chelator) was inferred from the increase in molecular weight as shown by MALDI-TOF analysis to be approximately 1:0.5 (DTPA-B11-nanobody, 60 min reaction) and ~1:0.9 (DTPA-B11-IgG, 30 min reaction) (Figure S3). The smaller size of the B11-nanobody fragment results in less available lysine groups for conjugation and a result requires a longer reaction time or higher molar excess of chelator to achieve a similar conjugation ratio as the full-length B11-IgG.

### 3.3. Radiolabeling

After 30 min of radiolabeling at room temperature, an RCY of 94.8 ± 3.1% (*n* = 3) for [^44^Sc]Sc-B11-IgG and 73.6 ± 12.1% (*n* = 3) for the [^44^Sc]Sc-B11-nanobody was achieved. Both compounds were purified further using a PD-10 column to achieve >99% RCP. The radiolabeled compound remained at the origin (R_f_ = 0) and free ^44^Sc traveled with the solvent front (R_f_ = 1), representative scans are included in Supplementary Figure S4. The purified [^44^Sc]Sc-B11-IgG and [^44^Sc]Sc-B11-nanobody had an apparent molar activity (A_m_) at end of synthesis of 6.3 ± 3.2 GBq/µmol and of 5.1 ± 2.8 MBq/µmol, respectively (*n* = 3)

### 3.4. SDS-PAGE and Autoradiography

**Figure 1:**
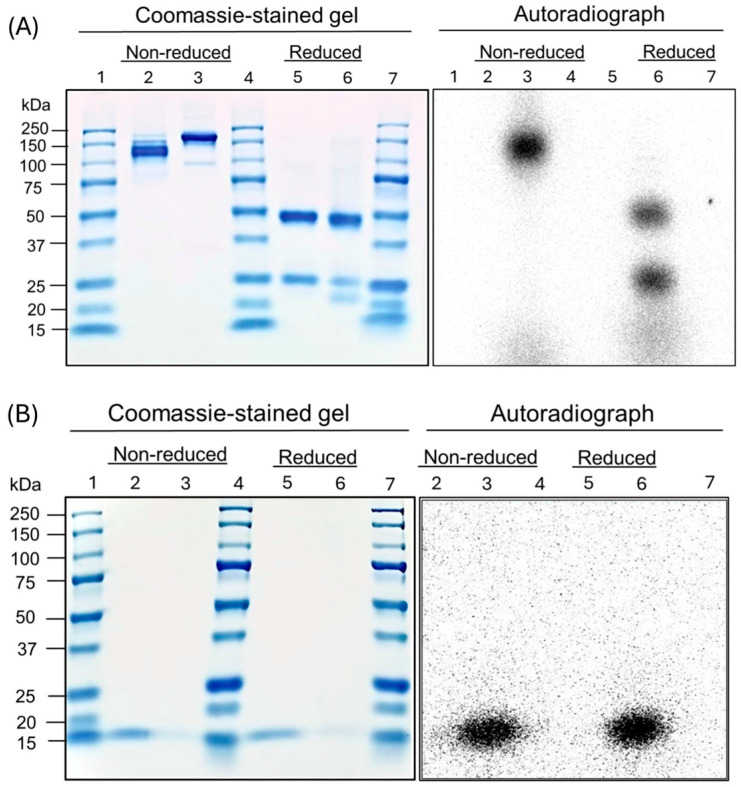
Coomassie-blue stained SDS-Page and autoradiography of non-radiolabeled and ^44^Sc-labeled (**A**) B11-Nanobody; where Lane 1: Protein marker marker (10-250 kDa, BioRad cat #1610373), Lane 2: B11-nanobody non-reduced, Lane 3: [^44^Sc]Sc-B11-nanobody non-reduced, Lane 4: Protein marker (2-250 kDa, BioRad cat #1610377), Lane 5: B11-nanobody reduced, Lane 6: [^44^Sc]Sc-B11-nanobody reduced, Lane 7: Protein marker (2-250 kDa, BioRad cat #1610377). (**B**) B11-IgG, where Lane 1: Protein Marker (2-250 kDa, BioRad cat #1610377), Lane 2: Lane 2: B11-IgG non-reduced, Lane 3: [^44^Sc]Sc-B11-IgG non-reduced, Lane 4: Protein marker marker (10-250 kDa, BioRad cat #1610373), Lane 5: B11-IgG reduced, Lane 6: [^44^Sc]Sc-B11-IgG reduced, Lane 7: Protein marker (10-250 kDa, BioRad cat #1610373).

SDS-PAGE of the proteins and their radiolabeled conjugates demonstrated primary bands at the expected molecular weights at ~150 kDa for B11-IgG (Figure 1A) and ~15 kDa for B11-nanobody (Figure 1B). For the B11-IgG, a shift in the molecular weight of the primary band was noticeable, likely due to the addition of the DTPA chelator. A minor band is also visible at ~100 kDa, suggesting some possible degradation. Additionally, the autoradiography of [^44^Sc]Sc-B11-IgG demonstrated a radiolabeled impurity of ≤10 kDa (Figure 1A). Encouragingly, the [^44^Sc]Sc-B11-nanobody showed no radiolabeled impurities (Figure 1B).

### 3.5. Stability of Radiotracers in Serum

**Figure 2:**
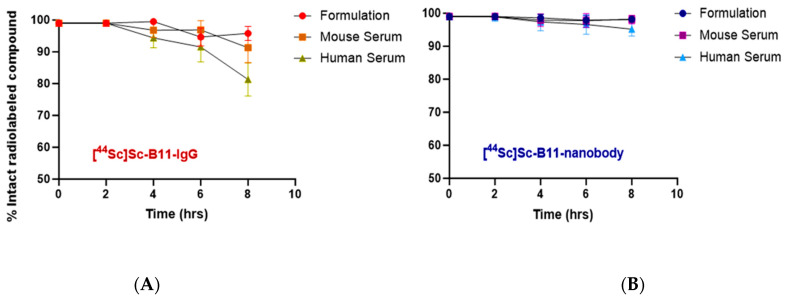
Stability of [^44^Sc]Sc-B11-nanobody (**A**) and [^44^Sc]Sc-B11-IgG (**B**), at 37 °C with light agitation (500 rpm) as formulated, and in mouse and human sera.

The stability of the radiolabeled full-length antibody and nanobody was assessed in its original formulation (0.25 NaOAc buffer, pH 5.7–6.0) and in mouse and human sera, at 37 °C with light agitation (500 rpm). As summarized in Figure 2, the percentage of intact [^44^Sc]Sc-B11-IgG after 8 h remained >95% in formulation but decreased to 91.3 ± 4.7% and 81.3 ± 8.3% in mouse and human sera, respectively, suggesting decomplexation or trans chelation in the presence of competing ions and/or natural ligands (Figure 2A, Table 4). Due to the larger molecular weight of the B11-IgG and thereby long plasma circulation time, stability over a longer period (>48 h) is of particular importance to allow for optimal uptake of the radioligand and clearance of background signal. For the [^44^Sc]Sc-B11-nanobody, the labeled compound was >98% intact after 8 h in formulation and >95% in human serum, encouraging further in vitro evaluation (Figure 2B, Table 4). The exact cause of the difference in stability between the radiolabeled B11-IgG and B11-nanobody, particularly in human serum, is unclear, but since both are conjugated to the same chelator and were synthesized with the same batch of ^44^Sc, this may reflect differences in the interactions of B11-IgG/B11-nanobody with serum proteins.

### 3.6. Cell Uptake

**Figure 3:**
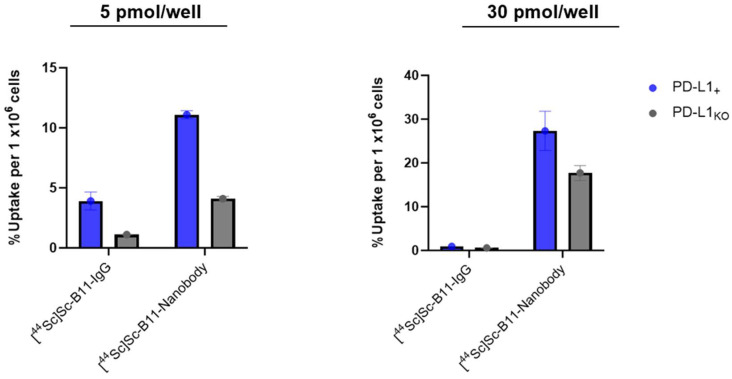
Cell uptake of [^44^Sc]Sc-B1-IgG and [^44^Sc]Sc-B11-nanobody at 5 pmol/well (**left**) and 30 pmol/well (**right**), in E0771 PD-L1+ breast cancer cells and E0771 PD-L1_KO_ cells.

After PD-10 purification, [^44^Sc]Sc-B11-IgG and [^44^Sc]Sc-B11-nanobody (30 pmol/well) were added to E0771 PD-L1 positive (+) and knockout (KO) cells. Higher uptake was observed in the PD-L1_+_ than PD-L1_KO_ cells for both the [^44^Sc]Sc-B11-IgG (0.91 ± 0.01 vs. 0.58 ± 0.05, *p* < 0.05) and [^44^Sc]Sc-B11-nanobody (27.33 ± 4.49 vs. 17.70 ± 1.71, *p* < 0.05) (Figure 3 and Table 5). Unexpectedly, the absolute uptake of [^44^Sc]Sc-B11-nanobody was significantly higher than the [^44^Sc]Sc-B11-IgG. However, there was also a significant amount of non-specific uptake in the PD-L1_KO_ cells (Figure 3). The relative uptake ratio (PD-L1+/PD-L1_KO_) was slightly higher for the [^44^Sc]Sc-B11-IgG than [^44^Sc]Sc-B11-nanobody (1.6 vs 1.5). This effect was replicated when the study was repeated with 5 pmol/well. The absolute uptake in PD-L1_+_ cells vs. PD-L1_KO_ was higher for [^44^Sc]Sc-B11-nanobody (11.1 ± 0.33 vs. 4.10 ± 0.19, *p* < 0.05), than the [^44^Sc]Sc-B11-IgG (3.91 ± 0.75 vs. 1.11 ± 0.02, *p* < 0.05) but the relative uptake (PD-L1_+_/PD-L1_KO_) was significantly higher for [^44^Sc]Sc-B11-IgG, due to high non-specific uptake of the [^44^Sc]Sc-B11-nanobody (Table 5).

## 4. Discussion

Noninvasive PET imaging-based assessment of PD-L1 expression is of high clinical value for better patient selection and treatment response rates to PD-L1 immunotherapies. Due to the slow clearance of full-length antibodies, smaller antibody fragments are attractive for use as radiolabeled molecular imaging probes if they maintain their selectivity and specificity. ^44^Sc has broad potential as a PET imaging radioisotope, particularly for use with peptides and antibody fragments. While 1,4,7,10-tetraazacyclododecane-1,4,7,10-tetraacetic (DOTA) is considered the gold standard for ^44^Sc chelation, this requires high temperatures (typically ≥ 80 °C). As such, acyclic chelators such as DTPA are better suited for use with heat-sensitive molecules such as antibodies/antibody fragments to allow for room-temperature radiolabeling. The stability of the [^44^Sc]Sc-B11-nanbody remained above 95% for up to 8 h, which encourages its use for further in vivo evaluation with ^44^Sc-labeled antibody fragments. Interestingly, the ^44^Sc-labeled B11-IgG showed poorer stability, remaining >95% intact in formulation up to 8 h but decreasing to ~80% in human serum. Since both the B11-nanobody and B11-IgG were conjugated with the same chelator and radiolabeled using the same batch of ^44^Sc and buffer/radiolabeling conditions, this could not be attributed to the complexation conditions. It is possible that the decomplexation is related to the degradation of the protein itself, but this was not probed further since the [^44^Sc]Sc-B11-IgG would not be a meaningful probe for in vivo evaluation due to the mismatch in the biological and physical half-life of the antibody and radiometal, respectively. The use of antibody fragments in place of a full-length IgG comes at the balanced trade-off between faster clearance but potential reduced specificity [20,34]. The radiolabeled B11-nanobody successfully showed significantly higher uptake in the PD-L1_+_ cells than the PD-L1_KO_. However, non-specific uptake was also observed, which negatively impacts its potential as a molecular imaging probe (potentially contributing to background signal and impacting the signal-to-noise ratio) and was, therefore, not advanced to further animal studies. The authors emphasize that these results are an evaluation of the particular nanobody studied and should not discourage future evaluation with other nanobodies for PD-L1 or otherwise. The suitability of the physical half-life of ^44^Sc with the biological half-life of nanobodies is highly encouraging for future evaluation with alternative nanobodies.

## 5. Conclusions

In this work,^44^Sc radiolabeled B11-nanobody and B11-IgG antibody were successfully synthesized with a final radiochemical purity > 99% and evaluated in an in vitro breast cancer model. The [^44^Sc]Sc-B11-nanobody was able to show higher uptake in PD-L1_+_ than PD-L1_KO_ cells, but the effect of lowered specificity was apparent in the high non-specific binding and overall uptake in the PD-L1_KO_ cells. For this reason, the ^44^Sc-labeled B11-nanobody was not advanced for further evaluation in an in vivo animal model. However, the high final radiochemical purity and stability data are encouraging for future evaluation of other nanobody-based PET probes radiolabeled with ^44^Sc.

## Figures and Tables

**Table 1:** MP-AES analysis conditions for Ca, Al and Fe analysis in final ^44^Sc elution fractions.

Element Wavelength (*λ*)	Nebulizer Flow Rate (L/min)	Viewing Position	Pump Speed	Stabilization Time
Ca (317.933 nm)	0.60	0	25 rpm	15 s
Al (396.152 nm)	0.95	0	25 rpm	15 s
Fe (371.993 nm)	0.65	0	25 rpm	15 s

**Table 2:** Summary of production activities (A) and yields (Y) of ^44^Sc at end-of-bombardment (EOB) for the ^nat^CaO target.

Current (µA)	Irradiation Time (min)	A_EOB_ (MBq)	Y_EOB_ (MBq/µAh)
15	15	49.95 ± 1.48 (*n* = 2)	9.99 ± 0.30
20	30	138.75 ± 1.49 (*n* = 3)	13.87 ± 0.15
20	60	246.42 ± 5.92 (*n* = 3)	12.32 ± 0.30
30	60	354.09 ± 8.51(*n* = 5)	11.80 ± 0.28
40	30	257.15 ± 4.44 (*n* = 3)	12.86 ± 0.22
40	60	483.22 ± 7.41 (*n* = 31)	12.08 ± 0.19
40	120	943.13 ± 5.98 (*n* = 3)	11.79 ± 0.07

**Table 3:** Analysis of remaining bulk Ca material and other trace metals (Fe, Al) in collected final elutions of ^44^Sc using a two-step DGA + SCX ion-exchange column set-up. Apparent specific activity reported for 30 min 40 µA productions at end of purification (no decay correction), assuming no other contaminants.

Fraction	Ca (ppm)	Fe (ppm)	Al (ppm)	Volume (µL)	Activity (GBq)	Apparent A_s_ (GBq/µg)
1	14.7 ± 5.6	2.2 ± 1.0	3.4 ± 1.6	500	109.8 ± 15.5	10.8 ± 7.7
2	8.7 ± 1.7	1.0 ± 0.3	1.4 ± 0.8	500	31.3 ± 4.9	5.6 ± 3.7
3	6.0 ± 0.01	0.6 ± 0.01	<0.01	500	10.1 ± 4.5	3.1 ± 2.2

**Table 4:** Percentage of intact [^44^Sc]Sc-B11-nanobody and [^44^Sc]Sc-B11-IgG over 8 h at 37 °C with light agitation (500 rpm) as formulated, and in mouse and human sera.

Stability	0 h	2 h	4 h	6 h	8 h
[^44^Sc]Sc-B11-IgG (% intact)					
Formulation	>99	>99	99.5 ± 0.9	94.7 ± 2.8	95.8 ± 2.2
Mouse Serum	>99	>99	96.8 ± 2.3	96.8 ± 2.9	91.3 ± 4.7
Human Serum	>99	>99	94.4 ± 3.1	91.5 ± 4.7	81.3 ± 8.3
[^44^Sc]Sc-B11-Nanobody (% intact)					
Formulation	>99	>99	98.6 ± 1.1	97.9 ± 1.4	98.2 ± 0.9
Mouse Serum	>99	>99	97.9 ± 1.9	97.8 ± 2.1	98.1 ± 1.3
Human Serum	>99	98.9 ± 1.3	97.4 ± 2.7	96.6 ± 2.9	95.2 ± 2.1

**Table 5:** Summary of cell uptake data comparing % uptake (per 1 × 10^6^ cells) in PD-L1_+_ and PD-L1_KO_ cells and the relative uptake ratio.

Compound	Apparent Molar Activity (A_m_) (GBq/µmol)	% Uptake (PD-L1_KO_)	% Uptake (PD-L1+)	*p*-Value	Uptake Ratio (+/_KO_)
		5 pmol/ well			
[^44^Sc]Sc-B11-IgG	4.6	1.11 ± 0.02	3.91 ± 0.75	4 × 10^−4^	~3.5
[^44^Sc]Sc-B11-nanobody	2.4	4.10 ± 0.19	11.1 ± 0.33	0.03	2.7
		30 pmol/ well			
[^44^Sc]Sc-B11-IgG	2.3	0.58 ± 0.05	0.91 ± 0.01	0.003	~1.6
[^44^Sc]Sc-B11-nanobody	6.9	17.70 ± 1.71	27.33 ± 4.49	6 × 10^−6^	~1.5

## Data Availability

All data generated in this study are presented in the work.

## Supplementary Materials

The following supporting information can be downloaded at: https://www.mdpi.com/article/10.3390/pharmaceutics17060796/s1, Figure S1: fragmentation of ^nat^CaCO3 and etching of the degrader foil at lo beam parameters (left) while ^nat^CaO target remained intact up to 40 µA for 2 hrs; Figure S2: Microimpurities present in ^nat^CaO target bombarded with 11.7 MeV protons to produce ^44^Sc (1157 keV). γ-spectrum shown two weeks post production (right) to clearly visualize radioimpurities of ^44m^Sc, ^43^Sc, ^47^Sc, ^48^Sc; Figure S3: MALDI-TOF plots of (A) B11-IgG, (B) DTPA-B11-IgG, (C) B11-nanobody and (D) partially conjugated (~46%) DTPA-B11-nanobody; Figure S4: radio-TLC scans of the radiolabeled proteins before and after purification with PD-10 desalting column; Table S1: Analysis of standard solutions containing Ca and Al at varying ratios, with and without 1 mg/mL CsNO_3_.

## Abbreviations

The following abbreviations are used in this manuscript:

PD-L1	Programed Death Ligand 1
PD-1	Programed Death 1
PET	Positron Emission Tomography
TRT	Targeted Radionuclide Therapy
SBR	Signal to Background Ratio
MP-AES	Microwave Plasma Atomic Emission Spectroscopy
AUC	Area Under the Curve
SDS-PAGE	Sodium Dodecyl Sulfate—PolyacrylAmide Gel Electrophoresis
HBSS	Hanks Buffered Salt Solution
