# Supplementary material for: Synthesis and In Vitro Evaluation of a Scandium-44 Radiolabeled Nanobody as a PD-L1 PET Imaging Probe
Source: Pharmaceutics. 2025 Jun 19;17(6):796. doi: 10.3390/pharmaceutics17060796 (PMC12196724; doi:10.3390/pharmaceutics17060796)
Supplement: Supplementary file 1 [file pharmaceutics-17-00796-s001.zip › pharmaceutics-3603108-supplementary.pdf]

Supplementary Materials

1. ^{44}Sc production and quality control

Two target materials, $^{nat}CaCO_3$ and ^{nat}CaO were evaluated in pressed powder pellet form with the ^{nat}CaO showing better stability under increasing beam current (10-40 μA) and time (10 min – 2 hrs) (Figure S1). As such ^{nat}CaO was implemented for routine production.

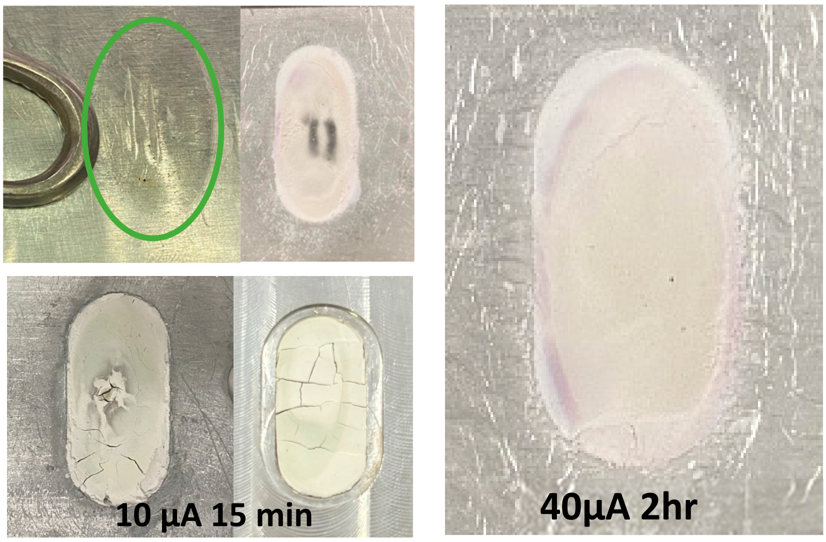

Figure S1: *fragmentation of $^{nat}CaCO_3$ and etching of the degrader foil at lo beam parameters (left) while ^{nat}CaO target remained intact up to 40 μA for 2 hrs.*

Table S1. Analysis of standard solutions containing Ca and Al at varying ratios, with and without 1 mg/mL $CsNO_3$.

**Sample**	**Ca (317.933 nm) (ppm)**	**Al (396.152 nm) (ppm)**
Blank	*0.00*	*0.00*
Standard 1	*0.10*	*0.10*
Standard 2	*10.00*	*1.00*
Standard 3	*100.00*	*10.00*
**1:1 ppm Ca:Al**	**1.13**	**1.02**
**10:1 ppm Ca:Al**	**10.01**	**0.96**
**100:1 ppm Ca:Al**	**98.47**	**1.02**
**100:0.1 ppm Ca:Al**	**98.52**	**0.10**
**1:1 ppm Ca;Al with $CsNO_3$**	**1.07**	**1.00**
**100:0.1 ppm Ca;Al with $CsNO_3$**	**94.43**	**0.10**

For analysis of remaining Ca in the eluted fractions used for radiolabeling and other trace metals (Fe and Al), 250uL aliquots of the samples were analyzed via MP-AES. To determine if an ionization suppressant was needed, control solutions ranging from Ca:Al ratios of 1:1 to 1000:1 were analyzed (Table S1). No suppression of the Al content or improvement in recovery was measured with the $CsNO_3$ containing samples at the concentrations tested.

Comparatively high activities of ^{44}Sc (γ=1157 keV) masked the presence of low levels of longer-lived impurities right after production, easily detectable >1 day after EOB (Figure S2). Their presence is inferred from their gamma spectrum but is not analyzed quantitatively in this work.

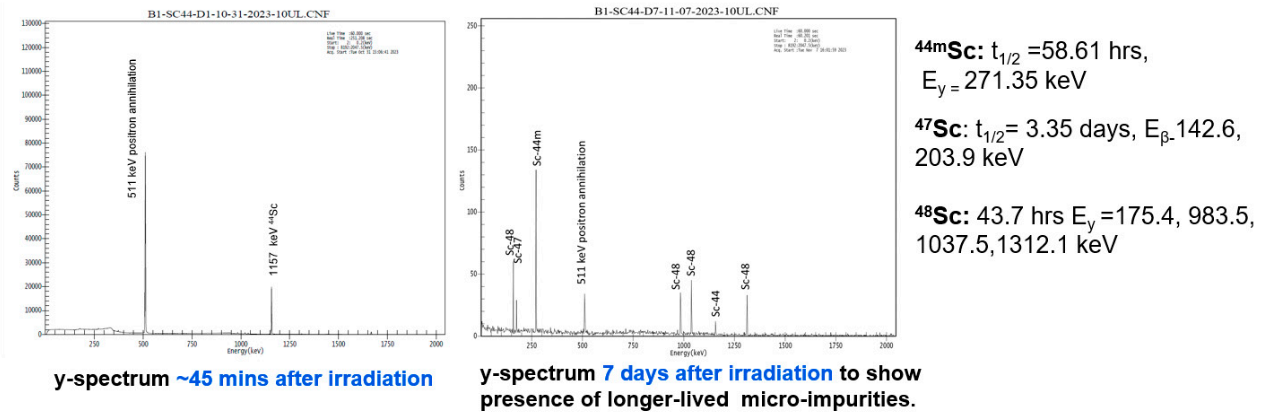

Figure S2: Microimpurities present in ^{nat}CaO target bombarded with 11.7 MeV protons to produce ^{44}Sc (1157 keV). γ-spectrum shown two weeks post production(right) to clearly visualize radioimpurities of ^{44m}Sc, ^{43}Sc, ^{47}Sc, ^{48}Sc.

2. Conjugation

The full-length B11-IgG and B11-nanobody were conjugated to S-2-(4 Isothiocyanatobenzyl)-diethylenetriamine pentaacetic acid (p-SCN-Bn-DTPA, MW= 649.9 Da) (Macrocylics, Texas, USA), a bifunctional acyclic chelator for ^{44}Sc, by adding a three-fold molar excess of chelator in 1X PBS at pH 9.0 and 37°C for 30 and 60 minutes, respectively. The conjugation ratio was determined via matrix assisted laser desorption ionization time-of-flight (MALDI-TOF) analysis, performed at the Mass Spectrometry Facility, School of Chemical Sciences, University of Illinois at Urbana Champaign. The chelation ratio was approximated by analyzing the shift in the molecular weight (Figure S3).

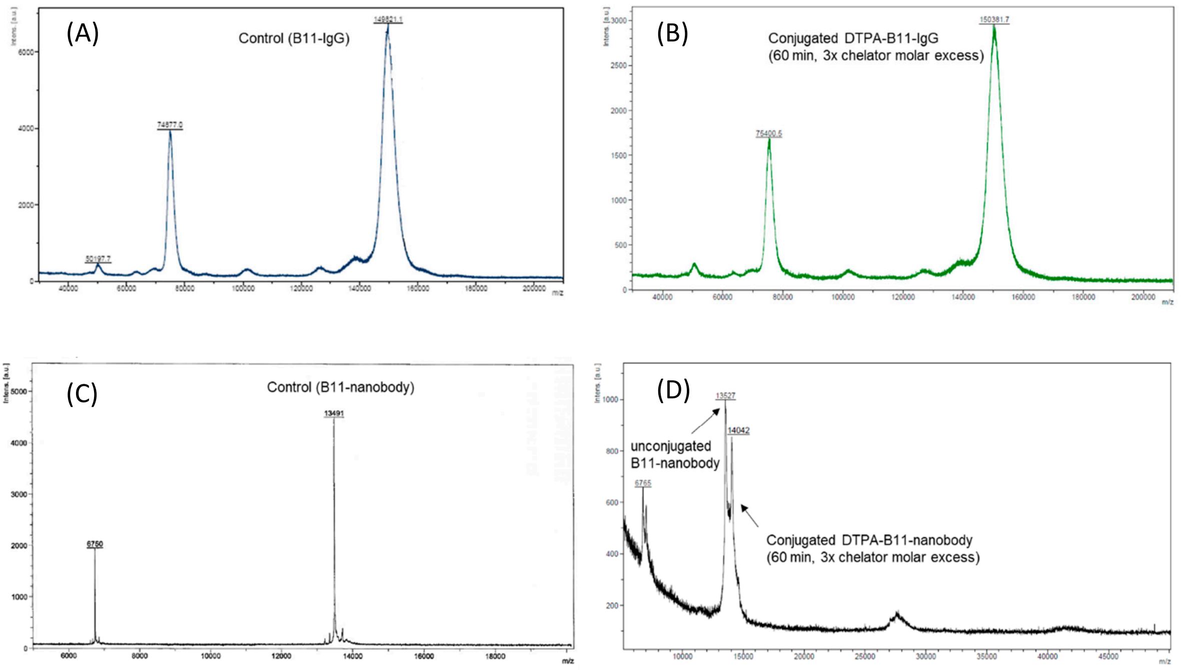

Figure S3: MALDI-TOF plots of (A) B11-IgG, (B) DTPA-B11-IgG, (C) B11-nanobody and (D) partially conjugated (~46%) DTPA-B11-nanobody

3. Radiolabeling

After 30 minutes of radiolabeling at room temperature, a RCY of 94.8 ± 3.1% (n=3) for [^{44}Sc]Sc-B11-IgG and 73.6 ± 12.1% (n=3) for [^{44}Sc]Sc-B11-nanobody. Both compounds were purified further using a PD-10 column purification to achieve >99% RCP. The radiolabeled compound remained at the origin (R_f=0) and free ^{44}Sc travelled with the solvent front (R_f=1), representative scans are shown in Figure S4**.**

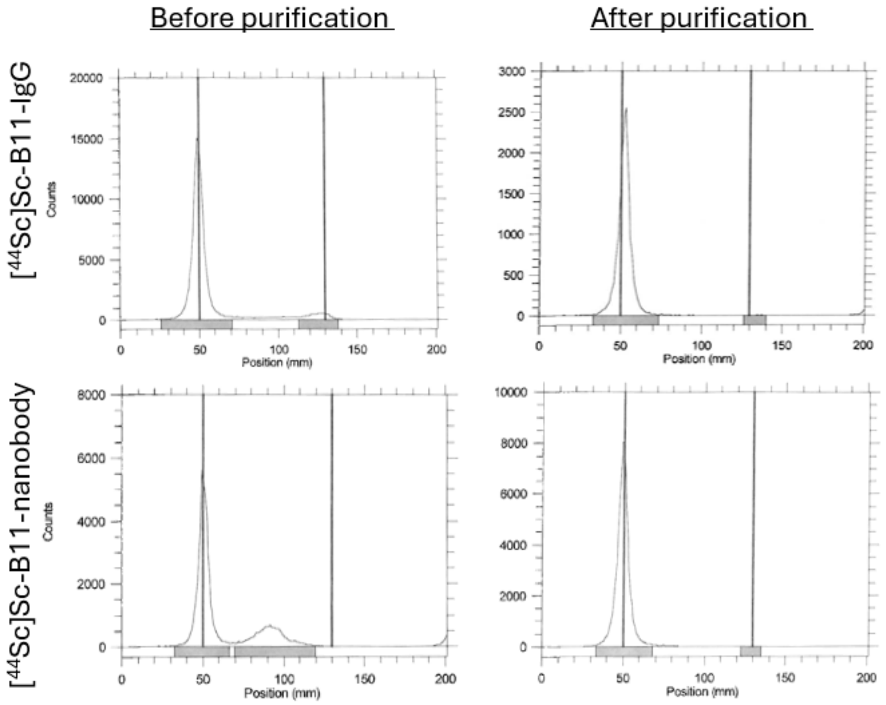

Figure S4: radio-TLC scans of the radiolabeled proteins before and after purification with PD-10 desalting column.
